# Digital Exclusion Among Mental Health Service Users: Qualitative Investigation

**DOI:** 10.2196/11696

**Published:** 2019-01-09

**Authors:** Ben Greer, Dan Robotham, Sara Simblett, Hannah Curtis, Helena Griffiths, Til Wykes

**Affiliations:** 1 Department of Psychology Institute of Psychiatry, Psychology and Neuroscience King's College London London United Kingdom; 2 National Institute for Health Research Maudsley Biomedical Research Centre South London and Maudsley National Health Service Foundation Trust London United Kingdom; 3 The McPin Foundation London United Kingdom

**Keywords:** digital exclusion, digital divide, digital inequality, technology, eHealth, mental health, social exclusion, mobile phone

## Abstract

**Background:**

Access to internet-enabled technology and Web-based services has grown exponentially in recent decades. This growth potentially excludes some communities and individuals with mental health difficulties, who face a heightened risk of digital exclusion. However, it is unclear what factors may contribute to digital exclusion in this population.

**Objective:**

To explore in detail the problems of digital exclusion in mental health service users and potential facilitators to overcome them.

**Methods:**

We conducted semistructured interviews with 20 mental health service users who were deemed digitally excluded. We recruited the participants from a large secondary mental health provider in South London, United Kingdom. We employed thematic analysis to identify themes and subthemes relating to historical and extant reasons for digital exclusion and methods of overcoming it.

**Results:**

There were three major themes that appeared to maintain digital exclusion: a perceived lack of knowledge, being unable to access the necessary technology and services owing to personal circumstances, and the barriers presented by mental health difficulties. Specific facilitators for overcoming digital exclusion included intrinsic motivation and a personalized learning format that reflects the individual’s unique needs and preferences.

**Conclusions:**

Multiple factors contribute to digital exclusion among mental health service users, including material deprivation and mental health difficulties. This means that efforts to overcome digital exclusion must address the multiple deprivations individuals may face in the offline world in addition to their individual mental health needs. Additional facilitators include fostering an intrinsic motivation to overcome digital exclusion and providing a personalized learning format tailored to the individual’s knowledge gaps and preferred learning style.

## Introduction

Internet use is near-ubiquitous in the United Kingdom with 80% of adults reporting using the internet daily [1]. The number of UK adults reporting having never accessed the internet is also declining, from 11.4% and 10.2% in 2015 and 2016, respectively [2,3], to 9% in 2017 [4]. Despite these decreases, a minority of individuals remain digitally excluded. There are inconsistent definitions of digital exclusion [5], but it may be broadly defined as being unable to access or use internet-enabled technology and Web-based services [6].

Digital exclusion has been associated with material deprivation in the offline world. For example, Helsper [7] theorized that material deprivation in areas such as economic capital and sociocultural affiliations, mediated by individual factors such as access and skills, influences digital exclusion. In support of this model, Longley and Singleton [8] mapped material deprivation [9] onto geographical areas in England characterized by low levels of digital engagement and found that high material deprivation areas had lower levels of engagement in internet-enabled technology. Evidence from a recent international survey in Sweden and Britain also indicates that digital exclusion is becoming increasingly concentrated among vulnerable populations, including those who are socially isolated and unemployed [10]. In addition, factors such as old age [11,12] and living in rural areas [13,14] have previously been associated with digital exclusion.

Digital technology and services, including electronic patient record systems, self-monitoring technology, and Web-based therapies [15], have been proposed as pivotal components of planned improvements to mental health provision in the United Kingdom over the next 5 years [16]. These technologies and services have the potential to improve communication between service users and health care professionals, empower service users to more actively manage their health, and augment clinical decision making with real-time clinical information [17]. For these benefits to be realized, mental health service users must be able to access and use these systems.

Surveys conducted in the United Kingdom and the United States indicate that digital exclusion is more prevalent among those with mental health difficulties compared with those without [18,19]. Globally, numerous social determinants of health are associated with poorer mental health, including indicators of lower socioeconomic status such as poverty and employment status [20,21]. Consistent with theories of social and digital exclusion [7], people with mental health difficulties may be more likely to experience digital exclusion because they are also more likely to be socially excluded [22,23]. Evidence also suggests that despite technological developments [24], the factors associated with digital exclusion have been consistent over time. For example, in a sample of outpatients diagnosed with schizophrenia, depression, or anxiety disorders, only 36% reported having ever used the internet, with the primary barriers to internet use including financial costs, lack of skills or knowledge, cognitive difficulties, and access [25]. Ennis et al [26], using a survey of community mental health service users, reported that factors related to knowledge and access were associated with digital exclusion. In a 5-year follow-up of that study, Robotham et al [6] reported that while self-reported rates of digital exclusion had declined, barriers to inclusion still existed. Consistent with Borzekowski et al [25] and Ennis et al [26], key barriers included a lack of knowledge, skills, and financial resources.

To ensure all mental health service users can reap the benefits of digital services for health care and in daily life, it is important to understand the factors that lead to the exclusion of some, as this may help identify specific targets for intervention. Due to the pace of development in internet-enabled technology and Web-based services, it is important to gain a contemporary understanding of why some individuals remain excluded. Qualitative approaches may offer a greater depth of understanding of how the barriers discovered in the surveys emerge and interact to cause digital exclusion. The aim of this study was to explore reasons for digital exclusion among mental health service users and potential facilitators to overcome it.

## Methods

### Design

This was an exploratory qualitative study. We employed semistructured interviews using a topic guide. Service users and carers who required assistance with accessing and using internet-enabled technology and services attended a computer skills session and reviewed all study materials and measures. Members of the research team facilitated these sessions. We obtained ethical approval from the North West-Haydock Research Ethics Committee (16/NW/0792). The Feasibility and Acceptability Support Team for Researchers, a team of mental health service users and carers who have been specially trained to advise on research proposals and documentation, reviewed the study protocol, information sheet, and consent form for readability and acceptability.

### Participants

We recruited participants from a large secondary mental health provider in South London, covering a diverse geographical area including areas of high poverty and urban deprivation. The participant recruitment sites included outpatient services, a community center, and weekly computer skills workshops where we recruited 4, 11, and 5 patients, respectively. We conducted recruitment and analysis concurrently. During recruitment, four authors (BG, DR, SS, and HC) met to discuss emerging themes and participant characteristics and, subsequently, employed maximum variation sampling [27] to recruit individuals who differed from current participants in terms of demographics and clinical diagnoses. We stopped recruitment when data saturation, the point at which interviews stopped yielding new themes, appeared to have been reached [28].

The inclusion criteria were being a current user of mental health services, possessing the capacity to provide informed consent (determined by the researcher trained in taking informed consent), and being digitally excluded (determined through a screening questionnaire, see below). The exclusion criteria were having a diagnosis of dementia, which is an illness that has specific needs in relation to digital intervention [29], and being under the age of 18, as previous research suggests true digital exclusion is less likely in this age group [30].

### Screening Questionnaire

The screening questionnaire assessed potential participants’ access and confidence with internet-enabled technology to determine whether they were digitally excluded (see Table 1). There was one question asking participants whether anything stopped them from using the internet. We considered participants who answered yes to at least one of these items as digitally excluded. Potential participants who did not indicate any barriers or difficulties using the internet were considered ineligible to participate.

### Interview Topic Guide

We based our interview topic guide (see Multimedia Appendix 1) on Robotham et al [6]. This began with an initial exploration of participants’ familiarity with the internet (eg, “Have you ever used the internet?” and “In your own words can you describe what the internet is?”). This was a warm-up discussion for the remainder of the interview and provided the interviewer with an overall understanding of the participant’s level of digital exclusion. We followed this with a discussion of potential barriers they may have encountered, as identified by Robotham et al [6] (eg, financial, knowledge, or skills), and facilitators to overcome digital exclusion (eg, delivering support in a group format vs one-on-one).

### Procedure

Following successful screening for digital exclusion, participants took part in interviews conducted in a location of their choosing, including community centers (n=11), hospitals (n=5), and private interview rooms (n=4). The interviews were audio recorded, and if the participant did not provide consent for audio recording (n=1), the researcher transcribed the participant’s responses in summary during the interview. The participants granted consent to use their electronic health records to obtain their demographic characteristics and diagnoses.

### Thematic Analysis

We transcribed interviews verbatim with personally identifiable content omitted and used NVivo 11 software (QSR International, Melbourne, Australia) [31] for thematic analysis [32]. Initially, one author (BG) read and reread the transcripts, generating an initial list of codes based on the semantic content of the transcripts. The authors collated these into a list of candidate themes and subthemes through consultation with each other. Another author (HG) then independently double-coded the transcripts using the candidate themes as a framework. BG and HG discussed any discrepancies between their codes until they reached a consensus and revised the themes into their final format.

**Table 1:** Screening questionnaire assessing access and confidence with internet-enabled technology (N=20).

Question		Responses, n (%)
**Which of the following items are you familiar with?**		
	Computer	3 (15)
	Computer and smartphone	4 (20)
	Computer, tablet, and smartphone	13 (65)
**Which of the following items do you own?**		
	Computer	7 (35)
	Computer and smartphone	4 (20)
	Tablet and smartphone	1 (5)
	None of them	8 (40)
**Which of the following items do you have access to?**		
	Computer	13 (65)
	Computer and smartphone	5 (25)
	Computer, tablet, and smartphone	1 (5)
	None of them	1 (5)
**Is there anything that stops you from using the internet?**		
	Lack of knowledge about how to use technology	17 (85)
	Not wanting to use technology	5 (25)
	Lack of available technology	10 (50)
	Lack of places to access technology	8 (40)
	Fear of technology	10 (50)
	Lack of credit or money	12 (60)
	Security concerns	13 (65)
	Other (participant-reported “confusion”)	1 (5)

## Results

### Participants

We approached 36 individuals, of whom 20 provided informed consent and were interviewed. The final sample contained 13 men and 7 women with a mean age of 56.7 years (SD 11.3; range 39-80). Additional participant characteristics are shown in Table 2. On average, participants had been in contact with mental health services for 19 years.

### Factors Maintaining Digital Exclusion

#### Knowledge

A perceived lack of knowledge was one of the most commonly reported barriers to engagement with internet-enabled technology. Participants reported confusion over how to use Web-based services, in addition to the internet-enabled technology itself:

What’s the reason I haven’t used the internet yet? I just can’t get my head around it. I don’t seem to be able to understand it so, I’ve put it on the back burner since I can’t understand it, you know?

P019, man, 57

I just think God; how do they do that? You know, but where is it? How do you get it? How do you set it up? I suppose it is on the computer, is it? And you are on your phone, and all that, and I just think, you know. I don’t know what half the things are, when they say “oh, do this, do that.” I’m completely ignorant.

P014, woman, 71

Some participants also expressed uncertainty regarding potential sources of help for overcoming their digital exclusion, with those who did suggest sources of help (n=11) typically suggesting library services:

I don’t know I don’t know where to ask for help, I don’t.

P004, woman, 57

Maybe the library, not sure? [laughter] perhaps the library?...Well I would ask if somebody can help me on the internet I suppose. Some of them are quite literate, computer literate and a lot more than I am so. But apart from that no I have no idea, but I mean if there are people I need to know [laughter].

P010, woman, 52

#### Personal Circumstances

Participants reported a range of personal circumstances as reasons for their digital exclusion. Perceived financial barriers were evident, including being unable to afford internet-enabled devices and accompanying services such as broadband:

Yeah if I could use a computer from home yeah, it’s about affording it as well you know what I mean all the internet, broadband and stuff like that, yeah yeah.

P007, man, 52

I’m on benefits you know, that’s it really…Yeah, once I have bills and all that and…event…and this that…I have nothing left for myself. And once you budget food out as well…you know what I mean?

P020, man, 61

Participants also reported barriers relating to their living situation, though it was unclear to what extent this may be related to financial costs:

Because I because I can’t get...Wi-Fi...at home so I have to go to the library with my Chromebook.

P004, woman, 57

Well, because I live in a house which is split into studios, and they don’t allow you to have an internet connection there you’re not allowed to get Sky or broadband brought into the building.

P017, man, 39

#### Mental Health

Participants reported how their mental health difficulties, specifically psychosis, impacted on their digital exclusion. This included relapses and hallucinations preventing them from being able to use internet-enabled technology and forgetting how to use the technology. These memory difficulties also appeared to have hindered previous attempts to overcome digital exclusion:

I’m not sure whether I’d be able to use a computer if I had a relapse because the last time I was all over the place it was terrible.

P002, man, 60

Cos I’m schizophrenic you see so, some days I might be hearing voices and so I might not be able to or things like that, you know.

P006, man, 59

Because as I’m a psychotic patient you know I find it very difficult to remember things.

P004, woman, 57

No, because they used to—we used to—do a group here on computers and this is like 2012 and once a week, but every week I would like forget what I learnt cos we were only once a week, once a week is not good enough to do them sort of things you know?

P019, man, 57

In addition to the impact of mental health difficulties themselves, periods of time spent in inpatient care were also reported to be detrimental to participants’ awareness of advances in technological development:

Well I think I think it’s mainly the gaps, when you’re mentally ill it’s not like popping in to hospital for, cos I’ve had an operation as well.Wherethey’re really keen to keep you, sometimes you’re ill for months and you know you get gaps and stuff and as I say things just move on so fast. There was Twitter and things like that and apps I’ve no idea what all those things are.

P012, woman, 57

**Table 2:** Participant characteristics (N=20).

Characteristics		Participants, n (%)
**Primary diagnosis**		
	Psychosis	10 (50)
	Affective disorder	4 (20)
	Personality disorder	1 (5)
	Eating disorder	1 (5)
	Other	4 (20)
**Highest education achieved**		
	Secondary but not exam qualifications	4 (20)
	Secondary (ordinary level or General Certificate of Secondary Education equivalent)	6 (30)
	Secondary (Advanced level equivalent)	2 (10)
	Vocational education or college	2 (10)
	Higher-level qualification (eg, university degree or professional qualification)	6 (30)
**Current occupation** **or employment**		
	Employed part-time	1 (5)
	Unemployed	9 (45)
	Student	2 (10)
	Volunteering	5 (25)
	Retired	3 (15)
**Ethnicity**		
	White British	7 (35)
	Black British	4 (20)
	Asian	2 (10)
	White other	2 (10)
	Black other	4 (20)
	Mixed	1 (5)

### Overcoming Digital Exclusion

#### Motivation

There was variation in participants’ motivation to overcome their digital exclusion. Some participants reported that digital exclusion did not negatively impact their lives and, therefore, did not express a desire to overcome it. For some who did report a desire to overcome their digital exclusion, it appeared that a perceived external pressure was an underlying factor:

It’s not actually providing anything for me, it’s just a machine it’s not providing anything for me.

P006, man, 59

I find that otherorganizationstoo, banks and so on, more and more indicating that the preferred way for theircustomersto deal with them is through the internet...and that is a steady pressure as it were and is a growing pressure…and we may get to a stage where people not using the internet are such a minority that they’re- they’re disregarded.

P015, man, 80

Age also appeared to be a moderating factor in participants’ motivation, with some older individuals being comfortable with digital exclusion. However, when asked whether efforts should be made to support older individuals in overcoming their digital exclusion, participants agreed that this population should not be overlooked:

Yes, I, I can easily say I would be open to that, whether I will actually grasp at it when the—when it was offered on the table in front of me…I’m not so sure, but it depends on what else was going on in my life

P015, man, 80

Yes, of course, yes absolutely. We’re out there, you know, we’re still alive and kicking, maybe sort of a bit slower—but we are out there.

P016, woman, 75

#### Personal Support Requirements

Participants expressed support for efforts to overcome digital exclusion that incorporated one-on-one and group support. Participants identified the social aspects of these approaches to support as key benefits:

Something positive to do somewhere positive to go. Don’t have to be stuck indoors all the time I can get out and meet people and learn new skills if you like.

P001, man, 47

Those kind of you know you can do a course online, mental health management and all this stuff online, to me it’s very isolating I prefer to be in a group and doing that kind of stuff rather than sitting at a computer doing it, so I do see a lot of the mental health kind of like workshops or courses or, you know, how to manage your mental health and all that and I just don’t even bother looking at them, cos I just know I’m not gonna do it.

P009, woman, 52

However, participants also expressed some concerns regarding group support, including the level of individual support that could be offered in a group format compared with one-on-one:

Well you wouldn’t be, the tutor wouldn’t necessarily be able to give you his or her undivided attention, if there’s a group.

P016, woman, 75

Where efforts to overcome digital exclusion may extend over multiple sessions, participants also voiced concerns regarding the level of commitment required and the potential consequences of missing a session:

I think for me in particular I think courses is something that you’ve got to kind of, you know you’ve got to commit to it I think for me it would be more like drop-in sessions on a particular subject. And more kind of one-to-one basis than than in a classroom…because especially with certain mental illnesses, mine in particular, I think if it’s a class I become quite anxious if I’m not able to attend it. And then going back to it after not attending would create a lot of anxiety for me.

P017, man, 39

## Discussion

### Factors Maintaining Digital Exclusion

This study identified three major themes maintaining digital exclusion among mental health service users: knowledge, personal circumstances, and mental health. Similar themes have been reported in previous surveys of digitally excluded mental health service users [6,25]. By employing interviews, this study extends these earlier findings by identifying the specific barriers that exist within these overarching themes. Participants in this study also reported additional barriers, including relapses, memory difficulties, and periods of time spent in inpatient care, not previously identified in past research in relation to mental health.

Participants reported that both material deprivation (eg, personal finances and living situation) and aspects of their mental health were barriers to engagement with internet-enabled technology. This means that efforts to overcome digital exclusion among individuals with mental health difficulties should address both the multiple deprivations that digitally excluded individuals may experience in the offline world and their specific mental health needs. For example, hallucinations and poor memory may negatively impact on an individual’s ability to engage and retain acquired skills in the future. So, understanding specific health needs may facilitate both short- and long-term digital inclusion. Gaps in knowledge and familiarity in people who have been isolated from developments in internet-enabled technology and services, such as those in inpatient services, should also be addressed. Providing supervised access to these technologies and services during periods of inpatient treatment may prevent these gaps arising, although this may not be required for everyone receiving inpatient mental health treatment. For example, people receiving treatment within acute mental health services, where the average length of stay is typically short, may experience a brief disruption in their access to internet-enabled technology but are unlikely to be completely excluded when returning to the community.

### Overcoming Digital Exclusion

The range of reported knowledge gaps suggests that a single umbrella approach to overcoming digital exclusion may not be effective because it presupposes an equal level of understanding and confidence. While some may benefit from assistance tailored specifically toward internet use, others may require fundamental instruction in operating internet-enabled technology. Formally evaluating individuals’ perceived confidence and competence would be beneficial to inform the scope and content of approaches to overcome digital exclusion. This would enable specific, individualized goals concerning technology and internet use to be set. Finding effective ways of ensuring digitally excluded individuals are aware of sources of support is also necessary.

Motivation was a key facilitator for overcoming digital exclusion but depended on a perceived disadvantage of being excluded. Without intrinsic motivation, engagement with programs to overcome digital exclusion will not be successful. For individuals expressing ambivalence about internet-enabled technology, techniques such as motivational interviewing [33,34] could be employed to foster this intrinsic motivation. There may be some instances, where digital exclusion does not exert a negative impact on the individual’s life, when efforts to overcome digital exclusion are not necessary or desired by the individual. In addition, older people are often thought to be unmotivated to use internet-enabled technology [11], but the findings of this study indicate that this is not always the case.

Personal support was a facilitator, but participants did not all value the same format of support. Efforts to overcome digital exclusion would benefit from an individually tailored learning approach. There was a preference for individual support, but if a one-on-one approach is not feasible, then small groups could facilitate individual support more readily than large groups. In addition, the risk of individuals falling behind owing to an absence or inability to engage should also factor into this learning approach. This could be mitigated by supplementary written materials or the option to refresh their learning, an option endorsed by multiple participants.

Participants reported financial barriers but did not specify what they believed the cost of internet-enabled technology and services to be. This should be explored, as individuals may overestimate this cost compared with the cost of available technology and services. Where financial barriers do exist, individuals could be signposted to free sources of internet-enabled technology and services, such as libraries and other public services. The option to supply these devices on a temporary or permanent basis could be explored but would need to be considered against the number of individuals requiring this support.

### Limitations and Future Research

Previous research highlights that digital inclusion is not always permanent, with some individuals who were previously digitally engaged subsequently disengaging [35]. There were 14 participants in this study who reported previously using the internet; however, this study did not compare whether the reasons for disengagement differ from those of never engaging with the internet or whether the consequences of each type of exclusion differ. Future research should, therefore, ensure that participants’ status as digitally naïve or digitally disengaged is identified, as there may be shared and divergent causes and consequences of both types of exclusion.

Reported difficulties with hallucinations are arguably specific to those with a diagnosis of psychosis, but other difficulties, such as amotivation and anhedonia, are shared across multiple diagnoses and may also impact on the ability to engage with internet-enabled technology and services. As our sample had 50% (10/20) participants with a psychosis diagnosis, this allowed both these specific and shared symptoms to be reported as affecting digital exclusion. Future research could benefit from a more comprehensive formulation of individuals’ difficulties beyond the level of diagnosis, to identify and explore the impact of specific clinical difficulties on digital exclusion.

We recruited 25% (5/20) participants from computer skills workshops, and they may have possessed a higher level of intrinsic motivation and knowledge than the other participants. This group allowed the exploration of these different potential motivational effects, and shared factors were identified between this group and the remaining group who had not experienced such training.

Future research should consider whether there are any potential variables, such as demographic characteristics, that may relate to digital exclusion. Exploring how these factors have historically impacted on digital exclusion could help to identify at-risk groups and inform personally tailored approaches to overcoming digital exclusion.

### Conclusion

This study identified three themes that maintain digital exclusion among mental health service users: knowledge, personal circumstances, and mental health. Efforts to support mental health service users to overcome digital exclusion must, therefore, address the multiple deprivations individuals may face in the offline world and their individual mental health needs. In addition to addressing wider societal issues related to finance and living circumstances, specific facilitators for overcoming digital exclusion include fostering an intrinsic motivation to overcome it and a personalized learning format tailored to individuals’ specific knowledge gaps and preferred learning style.

## Appendix

Multimedia Appendix 1 Topic guide used in participant interviews.
