# Supplementary material for: Digital Exclusion Among Mental Health Service Users: Qualitative Investigation
Source: J Med Internet Res. 2019 Jan 9;21(1):e11696. doi: 10.2196/11696 (PMC6329420; doi:10.2196/11696)
Supplement: Multimedia Appendix 1 [file jmir_v21i1e11696_app1.pdf]

Appendix A

Interview Topic Guide

Have you ever used the internet?

- when did you first use it?
- how often do you use it?
- what devices have you used to access the internet (did you use a computer, smartphone etc.)
- how easy or difficult did you find using this device for the internet?

In your words could you describe for me what the internet is?

Where have you been when you have used the internet (do you access it from home, libraries, internet cafes….)

- where do you prefer to use the internet? Why is that?

Can you tell me about the kind of things you use the internet for?

- ever used for healthcare; aware it can be used for healthcare?

Do you find using the internet interesting?

- Why do you say this (e.g. what in particular do you find interesting/ why aren't you interested?)

Have you found anything particularly difficult when trying to use the internet? (if so what, e.g. financial barriers, age barriers, privacy concerns…)

Do you feel as though you are ‘missing out’ on anything by not using the internet more?

-if so, what do you feel you are missing out on?

Have you ever had any help using the internet (e.g., computer skills classes, friends)

-Can you tell me about this (e.g. size of lesion/one-to-one, topics covered, length of time being helped…)

-Did you find this helpful, why?

Would you like to learn more about using the internet?

-What sorts of things would you like to learn?

What would be the best way of helping you to learn more about using the internet (e.g. class format or 1:1, topics covered, follow-up support, provision of devices?
